# The Prevalence of Gastroesophageal Reflux Disease (GERD) in H. pylori-Positive and -Negative Patients

**DOI:** 10.7759/cureus.72059

**Published:** 2024-10-21

**Authors:** Walid Alkeridy, Khalid Alanezi, Faisal K Alshehri, Mudafr Alkhedr, Mohammed A Albabtain, Musab Alamri, Rayan Jabaan, Abdulrahman Almugren, Majid Alsahafi, Saad S Alkhowaiter

**Affiliations:** 1 Department of Medicine, College of Medicine, King Saud University, Riyadh, SAU; 2 Department of Medicine, Geriatric Division, University of British Columbia, Vancouver, CAN; 3 General Administration of Home Health Care, Therapeutic Affairs Deputyship, Riyadh, SAU; 4 Division of Gastroenterology, Department of Medicine, Faculty of Medicine, King Abdulaziz University, Jeddah, SAU; 5 Department of Medicine - Gastroenterology, College of Medicine, King Saud University, Riyadh, SAU

**Keywords:** gerd, h. pylori, infection, patient, prevalence

## Abstract

Introduction: The presence of Helicobacter pylori (H. pylori) infection can influence gastric acid production. While both gastroesophageal reflux disease (GERD) and H. pylori are highly prevalent in the Saudi Arabian population, the relationship between these two conditions remains uncertain. This study aims to assess the association between GERD and H. pylori.

Methods: This was a cross-sectional study which included patients who underwent endoscopy with gastric biopsy to test for H. pylori. H. pylori status was assessed using histopathology. The GERD-Q questionnaire was used to ascertain the presence of GERD. Multivariate analysis was used to assess the relationship between H. pylori and GERD.

Results: A total of 352 participants were included, with a mean age of 45 years (±14.7) and 58% of them being female. 148 participants (42%) tested positive for H. pylori. 59 (39.9%) had GERD. GERD was identified in 39.9% of the H. pylori-positive group and 37.3% of the H. pylori-negative group. H. pylori was not significantly associated with GERD in the multivariate analysis (P = 0.726, OR = 1.089, 95% CI: 0.675 - 1.759).

Conclusion: There was no association between H. pylori infection and GERD in this Saudi Arabian population.

## Introduction

Gastroesophageal reflux disease (GERD) is a chronic gastrointestinal disorder characterized by the regurgitation of gastric contents into the esophagus [1-3]. GERD manifestations include heartburn, regurgitation chest pain, and difficulty swallowing [1]. Furthermore, H. pylori infection is a bacterial infection that can cause chronic gastritis, peptic ulcer disease, and gastric cancer [4,5]. Both GERD and H. pylori infection represent significant global health burdens. GERD affects up to 25% of the population in North America and Europe, while recent data from Saudi Arabia suggest an even higher prevalence, reaching up to 45%. Similarly, H. pylori infection remains highly prevalent in Saudi Arabia, with estimates indicating that up to 40% of the population may be affected [6-12]. Most previous studies have shown no association between H. pylori and GERD, while other literature has questioned this data. For example, a study in Wuhan reported a higher likelihood of GERD in H. pylori-positive patients, while an Indonesian study found a high prevalence of GERD despite low H. pylori infection rates. Additionally, some research found no significant impact of H. pylori on reflux esophagitis prevalence, and a case-control study even suggested a lower prevalence of H. pylori in GERD patients [13-16]. H. pylori and GERD have distinct pathophysiological backgrounds. The pathogenesis of GERD involves either increased esophageal exposure to gastric juice and/or a reduced threshold for epithelial damage and symptom perception. This intricate balance is influenced by anatomical and physiological factors that can either preserve or injure the esophageal lining. On the other hand, H. pylori disrupts stomach homeostasis by inducing inflammation through pro-inflammatory cytokines, affecting D cells, G cells, and parietal cells, ultimately leading to reduced acid production [17,18]. From a diagnostic standpoint, GERD is diagnosed through clinical symptoms, endoscopy, and pH monitoring, whereas H. pylori infection is detected using both invasive and non-invasive methods, including endoscopic biopsy and stool antigen tests [19,20]. Despite extensive research, the relationship between H. pylori infection and GERD remains unclear and controversial, with no consensus on whether a significant association exists between the two conditions [18,21-23]. Further investigation is essential to clarify the complex relationship between GERD and H. pylori, to better understand the underlying pathophysiology of their interaction, and to address public health concerns such as screening recommendations for H. pylori. Therefore, this study aims to determine the association between GERD and H. pylori infection in Saudi patients.

## Materials and methods

This is a cross-sectional study performed at King Khalid University Hospital (KKUH), King Saud University Medical City (KSUMC), King Saud University, a tertiary hospital in Riyadh, KSA. The study was carried out in accordance with the Declaration of Helsinki and approved by the Institutional Review Board of the College of Medicine, King Saud University, Riyadh, KSA (23-0371-IRB).

We included all consecutive participants who underwent endoscope and had gastric biopsy to assess for H. pylori between June 2022 to May 2023. We excluded any patient with gastric cancer, and treatment by subtotal gastrectomy, sleeve gastrectomy, gastric bypass surgery and those who refused to participate.

We collected data through a questionnaire that was completed through phone interviews. We explained the purpose of the study to all participants and included those who agreed to participate. 

The questionnaire inquiries about gender, age, height, weight, chronic diseases, and current smoking status. Moreover, we used the well-validated GERD questionnaire (GERDQ). The questionnaire asks participants to recall how frequently they had GERD symptoms throughout the previous week [24-26]. Its sensitivity has been demonstrated to be 65% and its specificity to be 71%. On a scale of 0 to 3, each of the six symptoms will be assigned a point based on their frequency. The resultant score will vary from 0 to 12, with a score of 8 or above being used to diagnose GERD [25,6]. We used the Arabic version of the Gastroesophageal Reflux Disease (Ar-GerdQ) to assess the severity and frequency of GERD symptoms [7-9,24]. The Ar-GerdQ underwent extensive validation and reliability testing to ensure its validity in Arabic-speaking populations [24]. The validation process included translation and cultural adaptation, following best practice protocols for forward and backward translation and pilot testing to ensure clarity and cultural relevance [24].

Study population

We contacted 707 individuals who had endoscopic histopathology H. pylori test in KSUMC, from the period of June 2022 to May 2023 in KKUH in Riyadh, Saudi Arabia. Out of these individuals, 355 participants were from the exclusion criteria and 352 participants were eligible and agreed to participate in this study.

Statistical analysis

The continuous variables are summarized using either the mean and standard deviation or the median with interquartile range, depending on the data distribution. We used percentages and counts to describe categorical variables. The association between categorical variables were analyzed using the Chi-square test. We used unconditional logistic regression models to assess the strength and direction of the association between H. pylori infection and GERD. The models calculated odds ratios (OR) and 95% confidence intervals (CI), to measure the likelihood of GERD presence in relation to H. pylori infection and other factors. We evaluated the goodness-of-fit for the logistic regression model by using the Hosmer-Lemeshow test, ensuring that the model adequately fit the observed data. The statistical significance was set at a p-value of less than 0.05. All the analyses were performed using IBM SPSS Statistics version 29.0.1.0 (IBM Corp., Armonk, NY, USA). A thorough evaluation of potential associations was conducted, taking into account various confounding factors to ensure the reliability of the results.

## Results

The sociodemographic characteristics of the study participants are presented in Table 1. This study included 352 individuals who underwent biopsy for histopathological confirmation of H. pylori. The average age of the participants was 45 years, with a standard deviation (SD) of ±14.7, and 58% of the participants were female. The mean BMI was 28.5±6.5. Among participants without GERD, the mean age was 44.36±14.77 years, and the mean BMI was 27.87±6.29. In contrast, participants with GERD had a mean age of 46.39±14.42 years and a mean BMI of 29.57±6.81. Additionally, one-third of the participants (113, 32%) reported drinking more than one cup of coffee per day, and only 59 (17%) were active smokers. Chronic diseases were also reported.

**Table 1:** Baseline Characteristics of Study Participants

Variables	
Male n (%)	148 (42%)
Female n (%)	204 (58%)
Age n (Mean±SD)	352 (45±14.17)
Height n (Mean±SD)	352 (163±9,8)
Weight n (Mean±SD)	352 (76±18.7)
Body mass index n (Mean±SD)	352 (28.5±6.5)
Diabetes n (%)	76 (22%)
Hypertension n (%)	59 (17%)
Dyslipidemia n (%)	66 (19%)
Heart failure n (%)	4 (1%)
Ischemic heart disease n (%)	8 (2%)
Current smoking status n (%)	59 (17%)
Coffee drink (>1) n (%)	113 (32%)
H. pylori result (positive) n (%)	148 (42%)
H. pylori result (negative) n (%)	204 (58%)

Table 2 summarizes the associations between GERD and key clinical factors, highlighting significant associations with current proton pump inhibitor (PPI) use and hypertension, while no significant associations were found with other clinical factors.

**Table 2:** Associations Between Gastroesophageal Reflux Disease (GERD) and Comorbid Conditions PPI: proton pump inhibitor

Characteristics	Diagnosis (GERD – No GERD) Pearson Chi-Square		
	Chi-square Value	df	P value
Body mass index (Average) (Under Weight or Normal or Overweight or Obese)	5.276a	3	0.153
Diabetes (No or Yes)	2.431a	1	0.119
Hypertension (No or Yes)	7.587a	1	0.006
Dyslipidemia (No or Yes)	1.072a	1	0.300
Heart failure (No or Yes)	0.232a	1	0.630
Ischemic heart disease (No or Yes)	2.019a	1	0.155
Current smoking status (No or Yes)	0.307a	1	0.580
Coffee drink (0 or 1 or 1>)	0.954a	2	0.621
Current PPI use (No or Yes)	52.063a	1	<0.001>
H. pylori result (+/-)	0.247a	1	0.619

The relationship between H. pylori test results and GERD diagnosis are shown in Table 3. The chi-square test shows that there is no statistically significant relationship between GERD and H. pylori test results (Pearson Chi-square = 0.247, p = 0.619). The eta value of 0.026 indicates a very weak relationship between the variables, implying that the presence of H. pylori has no significant impact on the diagnosis of GERD. Overall, the data from this sample do not support a strong link between GERD and H. pylori infection.

**Table 3:** The Association of Gastroesophageal Reflux Disease (GERD) and H. pylori Diagnosis

		H-pylori test		Total	Pearson Chi-Square	p value
		Negative	Positive		0.247	0.619
Diagnosis	No GERD	128 (62.7%)	89 (60.1%)	217		
	GERD	76 (37.3%)	59 (39.9%)	135		
Total		204 (58%)	148 (42%)	352		

Table 4 presents the results of a multivariate logistic regression analysis identifying risk factors for GERD. The analysis found no significant associations between GERD and H. pylori, in the unadjusted or adjusted model. 

**Table 4:** Multivariate Logistic Regression of Risk Factors for Gastroesophageal Reflux Disease (GERD) PPI: proton pump inhibitor

Variable	P value	Odds ratio	95% C.I (Lower-Upper)
Unadjusted odds ratio for H. pylori	0.619	1.116	(0.723-1.724)
Age	0.886	1.039	(0.615-1.754)
Body Mass Index	0.393	1.243	(0.755-2.045)
Current PPI use	<0.001	5.256	(3.243-8.517)
Hypertension	0.172	1.566	(0.823-2.982)
Current smoking status	0.435	0.766	(0.392-1.497)
H. pylori	0.726	1.089	(0.675-1.759)

## Discussion

In this study we aimed to determine the association between H. pylori infection and GERD among participants in Riyadh, Saudi Arabia to clarify the role of H. pylori in GERD pathogenesis. Our findings revealed that 42% of participants were H. pylori positive, with a GERD prevalence of 39.9% in this group compared to 37.3% in the H. pylori-negative group. However, the frequency of GERD did not significantly differ between those with and without H. pylori infection. Multivariate logistic regression analysis, which accounted for confounding factors such as age, BMI, smoking status, caffeine intake, and current PPI use, showed that H. pylori infection was not associated with GERD. These results support the hypothesis that there is no significant association between H. pylori infection and GERD, aligning with findings from other studies [14,27]. Examining the association between H. pylori infection and GERD could provide significant clinical insights into the pathophysiology of GERD. H. pylori infection is known to cause antrum-dominant gastritis, which disrupts the function of somatostatin-secreting D cells in the gastric antrum [28]. This disruption interrupts the normal feedback inhibition of gastrin release by luminal acid, leading to elevated gastrin levels and increased acid secretion, as seen in patients with H. pylori-associated duodenal ulcers [28]. Additionally, resolving this issue in an area with high prevalence of both conditions might have implications for policymakers in Saudi Arabia and countries with similar epidemiological characteristics to undermine screening for H. pylori in patients with GERD.

Our findings align with several previous studies that found no significant association between H. pylori infection and GERD. For instance, studies involving young, healthy Japanese volunteers, as well as a large cohort of 1,916 patients, reported no effect of H. pylori on GERD prevalence. Additionally, Grand et al. supported our results by indicating that H. pylori does not play a significant role in GERD development, while highlighting hiatus hernia as a more relevant risk factor [27,29-31]. However, other studies, including a study by Mari et al., reported a direct relationship between H. pylori and GERD, highlighting the ongoing controversy in the literature [16]. This contradictory finding by Mari et al. could be related to their study population being larger and from diverse backgrounds. Moreover, diagnostic ascertainment in our study was solely based on histopathological confirmation of H. pylori; a highly specific methods and considered the gold standard in making the diagnosis of H. pylori infection [32]. These factors could potentially explain the differences observed in the relationship between GERD and H. pylori between our studies. 

The association between GERD and H. pylori is complex as some studies have found a protective role of H. pylori against Barrett's esophagus and its progression to adenocarcinoma, which signals that H. pylori infection might be associated with a reduced risk of developing Barrett's esophagus and its malignant sequala [33]. Corpus-predominant or pangastritis associated with H. pylori infection is linked to reduced acid secretion secondary to local inflammation and increased levels of cytokines [34]. However H. pylori infection has been commonly accepted as risk factor for developing gastric adenocarcinoma [35,36]. Other studies, including one conducted in China, suggested that H. pylori eradication may increase esophageal acid exposure and, consequently, GERD symptoms, proposing H. pylori as a potential protective factor against GERD [37]. 

Our study found a strong association between GERD and PPI use, which is possibly the effect of confounding by indication. Significant suppression of gastric acid is associated with increased severity of H. pylori gastritis. A controlled trial showed the eradication of H. pylori infection in patients with reflux esophagitis receiving long-term acid suppression decreases inflammation and gastritis [38]. However, H. pylori eradication is believed to unmask GERD symptoms rather than directly causing GERD [39]. This find has important clinical implication to the personalized approach for people living with GERD and receiving long-term PPIs. Considering these findings, European guidelines continue to support H. pylori testing in patients receiving long-term PPI treatment [38].

Future directions

Future studies should include larger, more diverse populations across multiple regions in Saudi Arabia to validate our findings and explore regional variations in the H. pylori-GERD relationship. Additionally, research is needed to investigate the relationship between long-term PPI use and H. pylori complications, particularly in patients with fundus-predominant gastritis, to better understand the pathogenesis of the heightened risk of complications following H. pylori eradication.

Strengths and limitations

To our knowledge this is the first study to examine the association between GERD and the presence of H. pylori in Saudi Arabia using the Arabic validated version of GerdQ. However, there are several limitations to consider. The cross-sectional design may introduce selection and recall biases. On the other hand, one of the limitations is the patient selection design, as the patients were only selected from the endoscopy unit. Additionally, the study’s single-center design limits the generalizability of our findings. Although patients were referred from various regions of Saudi Arabia, providing a broader perspective, the small sample size remains a limitation. Future studies with larger sample size and better design is needed. A cohort study in the general population with a sampling method representative of the population such as cluster sampling. Additionally, the use of a GERD questionnaire, with a sensitivity of 65% and specificity of 71%, may have influenced the accuracy of GERD diagnosis.

## Conclusions

This cross-sectional study conducted in Saudi Arabia revealed no statistically significant correlation between Helicobacter pylori infection and GERD. These findings indicate that H. pylori is unlikely to have a substantial impact on the development of GERD in this particular group of people. Additional research with larger populations and a different design, namely a cohort study, is needed to establish the relationship between H. pylori status and GERD. Other studies with the same descriptive design can only increase the power of the assumption but cannot prove causation. These observations could provide valuable information for the development of clinical guidelines and public health strategies in areas that have similar patterns of disease occurrence.
